# Clinical outcomes of stent-less percutaneous coronary intervention with intravascular lithotripsy and drug-coated balloon for severely calcified de novo coronary lesions

**DOI:** 10.1016/j.ijcha.2025.101707

**Published:** 2025-05-27

**Authors:** Toru Misawa, Tetsumin Lee, Takashi Ashikaga, Toshihiro Nozato, Masakazu Kaneko, Ryoichi Miyazaki, Masashi Nagase, Taishi Yonetsu, Tetsuo Sasano

**Affiliations:** a Department of Cardiology, Japanese Red Cross Musashino Hospital, Tokyo, Japan; b Department of Cardiovascular Medicine, Institute of Science Tokyo, Tokyo, Japan

**Keywords:** Severely calcified lesions, Intravascular lithotripsy, Drug-coated balloon, Percutaneous coronary intervention

Intravascular lithotripsy (IVL) followed by drug-eluting stent (DES) implantation has been proven to be a safe and effective strategy for severely calcified lesions [1]. However, achieving optimal lesion preparation with IVL in stent-less percutaneous coronary intervention (PCI) using drug-coated balloon (DCB) for severely calcified lesions may be challenging because calcification is not completely removed. Thus, this study aimed to evaluate the safety and the efficacy of DCB after IVL for the treatment of severely calcified lesions.

This retrospective, single-center study included 45 patients who underwent stent-less PCI with IVL and DCB for severely calcified de novo coronary lesions between July 2022 and May 2024. All lesions were treated with DCB as the final device. Target lesion failure (TLF) was defined as a composite of culprit lesion-related cardiac death, non-fatal myocardial infarction, and target lesion revascularization (TLR), and its incidence and associated factors were assessed. Representative cases of stent-less PCI with IVL and DCB were shown in Fig. 1A and 1B. This research was conducted in compliance with the Declaration of Helsinki for the investigation of human beings and was approved by the Institutional Ethics Committee on Human Research of Japanese Red Cross Musashino Hospital.

**Fig. 1:**
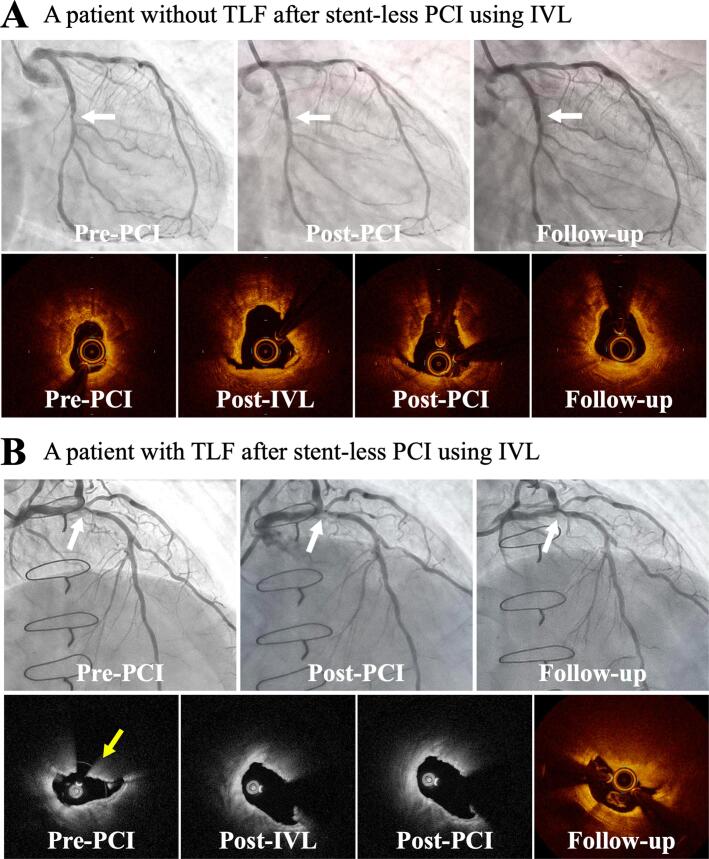
Representative cases of stent-less PCI with IVL and DCB; (A) A patient without TLF and (B) a patient with TLF. The white arrows indicate target lesions. The yellow arrow indicates calcified nodule. DCB, drug-coated balloon; IVL, intravascular lithotripsy; PCI, percutaneous coronary intervention; TLF, target lesion failure.

Baseline characteristics were shown in Supplemental Table 1. In a total 45 patients, the median age was 74 (interquartile range [IQR], 65–81) years, and 73.3 % were male. Most patients (N = 37, 82.2 %) presented with chronic coronary syndrome and 15.6 % of patients had end-stage renal failure requiring hemodialysis. All PCI procedures were performed under intravascular imaging guidance (optical coherence tomography [OCT]; 40, intravascular ultrasound [IVUS]; 5). Angiographic and IVUS/OCT findings were shown in Supplemental Table 2. Despite a calcium score of ≥ 3 on OCT or IVUS in all lesions [2], calcium fractures were observed in 84.4 % of patients. No periprocedural complications or bail-out stent deployments were observed in this study (Supplemental Table 3).

At the median follow-up period of 392 (IQR, 206–503) days, TLF events occurred in 4 patients (8.9 %); non-fatal myocardial infarction in 1 patient, and TLR in 3 patients.

Longer calcium length was observed in patients with TLF than those without (18.2 [IQR, 13.0–24.0] mm vs. 8.2 [IQR, 5.2–13.6] mm, P = 0.02) (Supplemental Table 2). However, reference vessel diameter was not significantly different between the groups (3.1 [IQR, 2.7–3.7] mm vs. 2.7 [2.4–3.1] mm, P = 0.38). In Cox regression analysis, only calcium length was associated with TLF (hazard ratio, 1.15; 95 % confidence interval, 1.00–1.33, P = 0.05), whereas non-statistically significant differences were found in prevalence of renal insufficiency required hemodialysis, calcium angle and frequency of calcified nodule between the groups. (Supplemental Table 4).

To the best of our knowledge, this is the first study to demonstrate the prognosis of stent-less PCI with IVL and DCB for severely calcified de novo coronary lesions and the factors associated with TLF. Although DCB following IVL remains off-label, its efficacy has been demonstrated in a recent case report [3]. TLF occurred in 8.9 % of patients in the present study, which was comparable to the previous study reporting TLF after IVL with DES in 6.3 % of patients at 1-year follow-up [1], as well as another study reporting 9.5 % of TLF in patients undergoing OCT-guided stent-less PCI with DCB for de novo coronary lesions [4]. Although calcium modification by IVL acoustic pressure waves might lead the morphological changes in calcified plaque and increase the drug permeability, precise mechanism, including morphological changes of the effectiveness of IVL with DCB is unclear. Therefore, further studies are warranted to determine the mechanism of the effectiveness of IVL with DCB. There were several limitations in this study. First, this study is retrospective, single-center design with a small population size, which can cause an inevitable selection bias. Second, statistical significance for key findings was marginal, and multivariable analysis could not be performed due to the small sample size. Therefore, our results should be interpreted with caution. Third, long-term outcomes after stent-less PCI with IVL were not assessed in the present study. A larger, multi-center trial with longer follow-up periods is needed to provide further insight into long-term follow-up and generalizability of the present study findings. Fourth, more detailed intravascular imaging analysis before and after the procedure, which could help elucidate the mechanism of the effectiveness of IVL with DCB, was not included. Fifth, there was no comparator group of DES. Though TLF of our study was similar to past study of IVL with DES [1], future studies of IVL with DCB compared with DES were needed to validate the benefits of stent-less PCI more rigorously. However, our results suggest that stent-less PCI with IVL and DCB is an alternative treatment option for severely calcified lesions.

In conclusion, DCB following IVL strategy was feasible for severely calcified de novo coronary lesions. Further large-scale, controlled studies are needed to confirm the safety and effectiveness of this strategy.

## Sources of funding

None.

## CRediT authorship contribution statement

**Toru Misawa:** Writing – original draft. **Tetsumin Lee:** Writing – review & editing, Supervision. **Takashi Ashikaga:** Writing – review & editing, Supervision, Conceptualization. **Toshihiro Nozato:** Writing – review & editing. **Masakazu Kaneko:** Data curation. **Ryoichi Miyazaki:** Data curation. **Masashi Nagase:** Data curation. **Taishi Yonetsu:** Writing – review & editing. **Tetsuo Sasano:** Writing – review & editing.

## Declaration of competing interest

The authors declare that they have no known competing financial interests or personal relationships that could have appeared to influence the work reported in this paper.
