# Supplementary material for: Clinical outcomes of stent-less percutaneous coronary intervention with intravascular lithotripsy and drug-coated balloon for severely calcified de novo coronary lesions
Source: Int J Cardiol Heart Vasc. 2025 May 27;59:101707. doi: 10.1016/j.ijcha.2025.101707 (PMC12158476; doi:10.1016/j.ijcha.2025.101707)
Supplement: Supplementary Data 1 [file mmc1.docx]

Supplemental Table 1. Patient’s baseline characteristics.

|  | All  (N=45) | Non-TLF  (N=41) | TLF  (N=4) | P |  |
| --- | --- | --- | --- | --- | --- |
| Age, yrs | 74 (65−81) | 74 (66−81) | 67 (64−73) | 0.44 |  |
| Male gender | 33 (73.3) | 29 (70.7) | 4 (100.0) | 0.56 |  |
| Acute coronary syndrome | 8 (17.8) | 8 (19.5) | 0 (0.0) | 1.00 |  |
| Chronic coronary syndrome | 37 (82.2) | 33 (80.5) | 4 (100.0) |  |  |
| Diabetes mellitus | 20 (44.4) | 18 (43.9) | 2 (50.0) | 1.00 |  |
| Hypertension | 34 (75.6) | 31 (75.6) | 3 (75.0) | 1.00 |  |
| Dyslipidemia | 26 (57.8) | 24 (58.5) | 2 (50.0) | 1.00 |  |
| Previous myocardial infarction | 10 (22.2) | 8 (19.5) | 2 (50.0) | 0.21 |  |
| Previous PCI | 23 (51.1) | 21 (51.2) | 2 (50.0) | 1.00 |  |
| Current smoking | 17 (37.8) | 15 (36.6) | 2 (50.0) | 0.63 |  |
| Renal insufficiency required hemodialysis | 7 (15.6) | 5 (12.2) | 2 (50.0) | 0.11 |  |
| LVEF, % | 67 (50−74) | 67 (50−73) | 74 (6276) | 0.46 |  |
| Laboratory data | | | | | |
| eGFR, ml/min/1.73m^2^ | 54.7 (32.8−66.6) | 56.0 (34.2−66.6) | 26.0 (8.5−51.6) | 0.35 |  |
| LDL−C, mg/dl | 85 (70−117) | 88 (72−117) | 62 (39−96) | 0.25 |  |
| HbA1c, % | 6.1 (5.7−7.1) | 6.1 (5.7−7.0) | 6.7 (5.9−7.5) | 0.46 |  |
| BNP, pg/ml | 65 (27−145) | 67 (27−145) | 39 (31−1319) | 0.83 |  |
| Hemoglobin, g/dl | 13.4 (11.5−14.6) | 13.4 (11.5−14.5) | 14.0 (12.5−14.7) | 0.74 |  |

Data are presented as number (%), or median (25−75th percentile).

BNP, B−type natriuretic peptide; eGFR, estimated glomerular filtration rate; HbA1c, glycosylated hemoglobin; LDL−C, low−density lipoprotein cholesterol; LVEF, left ventricular ejection fraction; PCI, percutaneous coronary intervention; TLF, target lesion failure.

Supplemental Table 2. Angiographic and IVUS/OCT findings

|  | All  (N=45) | Non-TLF  (N=41) | TLF  (N=4) | P |
| --- | --- | --- | --- | --- |
| Target vessel | | | | |
| LMT | 1 (2.2) | 1 (2.4) | 0 (0.0) | 1.00 |
| LAD | 24 (53.3) | 21 (51.2) | 3 (75.0) |  |
| LCX | 7 (15.6) | 7 (17.1) | 0 (0.0) |  |
| RCA | 13 (28.9) | 12 (29.3) | 1 (25.0) |  |
| Pre−PCI QCA | | | | |
| Minimum lumen diameter, mm | 0.7 (0.5−1.0) | 0.7 (0.5−1.0) | 0.9 (0.8−1.0) | 0.31 |
| Reference vessel diameter, mm | 2.7 (2.4−3.2) | 2.7 (2.4−3.1) | 3.1 (2.7−3.7) | 0.38 |
| Diameter stenosis, % | 75.5 (65.3−83.1) | 75.5 (67.2−83.5) | 73.3 (59.5−81.6) | 0.45 |
| Lesion length, mm | 20.8 (15.7−28.5) | 20.2 (15.7−28.5) | 21.8 (17.7−24.5) | 0.87 |
| Post−PCI QCA |  |  |  |  |
| Minimum lumen diameter, mm | 2.1 (1.9−2.5) | 2.1 (1.9−2.5) | 1.9 (1.9−2.3) | 0.58 |
| Diameter stenosis, % | 21.9 (19.0−25.7) | 21.8 (19.0−25.2) | 26.4 (23.7−27.9) | 0.19 |
| IVUS / OCT | 5 (11.1) / 40 (88.9) | 5 (12.2) / 36 (87.8) | 0 (0.0) / 4 (100.0) | 1.00 |
| Pre−PCI MLA, mm^2^ | 1.7 (1.2−2.7) | 1.7 (1.2−2.7) | 1.8 (1.3−2.5) | 0.89 |
| Pre−PCI maximum calcium angle, ° | 276 (200−324) | 268 (198−309) | 326 (321−332) | 0.07 |
| Pre−PCI maximum calcium thickness, μm | 1050 (841−1200) | 1050 (852−1186) | 1077 (841−1350) | 0.53 |
| Pre−PCI calcium length, mm | 10.4 (5.4−14.0) | 8.2 (5.2−13.6) | 18.2 (13.0−24.0) | 0.02 |
| Calcified nodule | 10 (22.2) | 8 (19.5) | 2 (50.0) | 0.21 |
| Calcium score 3 / 4 | 14 (31.1) / 31 (68.9) | 14 (34.1) / 27 (65.9) | 0 (0.0) / 4 (100.0) | 0.29 |
| Calcium fracture | 38 (84.4) | 35 (85.4) | 3 (75.0) | 0.51 |
| Post−PCI MLA, mm^2^ | 4.5 (3.5−4.9) | 4.6 (3.7−5.0) | 3.8 (3.2−4.0) | 0.09 |

Data are presented as number (%), or median (25−75th percentile).

IVUS, intravascular ultrasound; LAD, left anterior descending coronary artery; LCX, left circumflex coronary artery; LMT, left main coronary trunk artery; MLA, minimum lumen area; OCT, optical coherence tomography; PCI, percutaneous coronary intervention; QCA, quantitative coronary angiography; RCA, right coronary artery; TLF, target lesion failure.

Supplemental Table 3. Procedural results.

|  | All  (N=45) | Non-TLF  (N=41) | TLF  (N=4) | P | |
| --- | --- | --- | --- | --- | --- |
| IVL |  |  |  |  | |
| 2.5mm | 21 (46.7) | 18 (43.9) | 3 (75.0) | 0.76 | |
| 3.0mm | 19 (42.2) | 18 (43.9) | 1 (25.0) |  |  |
| 3.5mm | 4 (8.9) | 4 (9.8) | 0 (0.0) |  |  |
| 4.0mm | 1 (2.2) | 1 (2.4) | 0 (0.0) |  |  |
| Total pulses | 80 (60−80) | 80 (60−80) | 80 (70−80) | 0.88 | |
| DCB diameter, mm | 3.0 (3.0−3.5) | 3.0 (3.0−3.5) | 3.0 (3.0−3.5) | 0.64 | |
| Total DCB length, mm | 30 (20−50) | 30 (20−50) | 30 (30−45) | 0.90 | |
| Procedural complications | | | | |  |
| Post−PCI dissection (Type D−F) | 0 (0.0) | 0 (0.0) | 0 (0.0) |  | |
| Transient slow flow | 0 (0.0) | 0 (0.0) | 0 (0.0) |  | |
| Bail-out stent deployment | 0 (0.0) | 0 (0.0) | 0 (0.0) |  | |

Data are presented as number (%), or median (25−75th percentile).

DCB, drug−coated balloon; IVL, intravascular lithotripsy; PCI, percutaneous coronary intervention; TLF, target lesion failure.

|  | Hazard ratio | 95% CI | P |
| --- | --- | --- | --- |
| Renal insufficiency required hemodialysis | 6.06 | 0.85−43.28 | 0.07 |
| Pre−PCI diameter stenosis, % | 0.98 | 0.92−1.04 | 0.50 |
| Pre-PCI reference vessel diameter, mm | 1.72 | 0.57−5.17 | 0.33 |
| Pre−PCI MLA, mm^2^ | 1.29 | 0.46−3.60 | 0.63 |
| Pre−PCI maximum calcium angle, ° | 1.02 | 1.00−1.04 | 0.10 |
| Pre−PCI maximum calcium thickness, mm | 1.00 | 1.00−1.01 | 0.64 |
| Pre−PCI calcium length, mm | 1.15 | 1.00−1.33 | 0.05 |
| Calcified nodule | 2.94 | 0.41−20.95 | 0.28 |
| Post−PCI diameter stenosis, % | 1.17 | 0.91−0.22 | 0.22 |
| Post−PCI MLA, mm^2^ | 0.38 | 0.13−1.09 | 0.07 |

Supplemental Table 4. Univariable Cox regression analysis of TLF.

CI, confidence interval; MLA, minimum lumen area; PCI, percutaneous coronary intervention; TLF, target lesion failure.
